# DNA and Protein Requirements for Substrate Conformational Changes Necessary for Human Flap Endonuclease-1-catalyzed Reaction*

**DOI:** 10.1074/jbc.M115.698993

**Published:** 2016-02-16

**Authors:** Sana I. Algasaier, Jack C. Exell, Ian A. Bennet, Mark J. Thompson, Victoria J. B. Gotham, Steven J. Shaw, Timothy D. Craggs, L. David Finger, Jane A. Grasby

**Affiliations:** From the ‡ Centre for Chemical Biology, Department of Chemistry, Krebs Institute, University of Sheffield, Sheffield S3 7HF, United Kingdom and; the § DNA:Protein Interactions Unit, School of Biochemistry, University of Bristol, Bristol BS8 1TD,United Kingdom

**Keywords:** circular dichroism (CD), DNA endonuclease, DNA repair, DNA replication, DNA-protein interaction, fluorescence resonance energy transfer (FRET), nucleic acid enzymology

## Abstract

Human flap endonuclease-1 (hFEN1) catalyzes the essential removal of single-stranded flaps arising at DNA junctions during replication and repair processes. hFEN1 biological function must be precisely controlled, and consequently, the protein relies on a combination of protein and substrate conformational changes as a prerequisite for reaction. These include substrate bending at the duplex-duplex junction and transfer of unpaired reacting duplex end into the active site. When present, 5′-flaps are thought to thread under the helical cap, limiting reaction to flaps with free 5′-termini *in vivo*. Here we monitored DNA bending by FRET and DNA unpairing using 2-aminopurine exciton pair CD to determine the DNA and protein requirements for these substrate conformational changes. Binding of DNA to hFEN1 in a bent conformation occurred independently of 5′-flap accommodation and did not require active site metal ions or the presence of conserved active site residues. More stringent requirements exist for transfer of the substrate to the active site. Placement of the scissile phosphate diester in the active site required the presence of divalent metal ions, a free 5′-flap (if present), a Watson-Crick base pair at the terminus of the reacting duplex, and the intact secondary structure of the enzyme helical cap. Optimal positioning of the scissile phosphate additionally required active site conserved residues Tyr^40^, Asp^181^, and Arg^100^ and a reacting duplex 5′-phosphate. These studies suggest a FEN1 reaction mechanism where junctions are bound and 5′-flaps are threaded (when present), and finally the substrate is transferred onto active site metals initiating cleavage.

## Introduction

**FIGURE 1.**
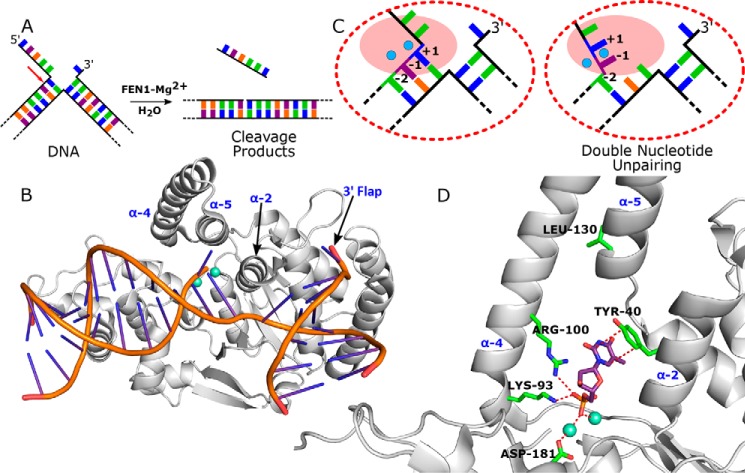
**FEN1 DNA bending and double nucleotide unpairing.** *A*, schematic of the FEN1 catalyzed hydrolysis of a double flap DNA yielding single-stranded DNA and double-stranded nicked DNA products. An *arrow* indicates the site of reaction. Each nucleobase is represented by a different color. *B*, hFEN1-product complex (Protein Data Bank code 3q8k) showing 100° bent DNA. *C*, schematic of double nucleotide unpairing proposed to position the scissile phosphodiester bond between the +1 and −1 nt on active site (*pink*) metal ions (*cyan*). *D*, cartoon representation of the active site in the FEN1-product structure (Protein Data Bank code 3q8k) showing the phosphate monoester of the unpaired −1 nt in contact with metal ions (*cyan*) and helical gateway (base α2-α4) and cap (top of α4 and α5) residues mutated in this study.

Flap endonuclease-1 (FEN1)^4^ is an essential component of the DNA replicative and repair apparatus and the prototypical member of the 5′-nuclease superfamily (1–5). FEN1 removes single-stranded DNA or RNA flaps formed during DNA replication and repair as a result of strand displacement synthesis. Flapped DNAs arising in this context (*e.g.* adjacent Okazaki fragments) are equilibrating (*i.e.* migrating) structures that can have differing lengths of 5′- and 3′-single-strands, because all flaps are complementary to the continuous DNA template. However, FEN1 only processes one flapped DNA conformer, a two-way DNA junction bearing a single nucleotide (nt) 3′-flap and any length of 5′-flap (see Fig. 1, *A* and *B*) (6–8). FEN1 then catalyzes specific phosphate diester hydrolysis of the flapped DNA 1 nt into the double-strand, ensuring that the product is nicked DNA (see Fig. 1*A*). This exquisite specificity is necessary for the fidelity and efficiency of DNA replication and repair, because nicked DNA can be joined immediately by DNA ligase.

Extensive work has led to models for the origins of FEN1 reaction specificity that rely on key DNA conformational changes for substrate recognition and reaction site selection. The first selection is for two-way junction DNAs and involves the substrate bending 100° to contact two separate double-stranded DNA binding sites (see Fig. 1*B*) (7–10). One of these duplex binding sites forms a substrate-induced binding pocket that can only accommodate a 1-nt 3′-flap, which explains the preference for substrates with a single 3′-flap nucleotide.

The second requirement of hFEN1 specificity excludes the reaction of continuous single-stranded DNAs (*e.g.* template strand during replication) or flaps with bound protein. Although controversial (11), the 5′-flap is thought to pass through a hole in the protein above the active site and bordered by the helical cap (top of α4 and α5) and gateway (base of α4 and α2) (see Fig. 1, *B* and *D*) (1, 8, 12–14). The final specificity requirement is for reaction 1 nt into duplex, which is the hallmark of the 5′-nuclease superfamily that also includes the DNA repair proteins EXO1, XPG, and GEN1 (1). This selectivity is believed to involve a local DNA conformational change at the terminus of the reacting duplex (5, 8, 15–17), whereby two gating α-helices (bases of α2 and α4) appear to prevent access of duplex DNAs to the active site (8). It is proposed that the last two 5′ nucleotides of the reacting duplex unpair to place the scissile phosphate diester bond on the catalytic metal ions (see Fig. 1, *C* and *D*).

Although the overall conformational changes that FEN1 substrates must undergo before reaction have been deduced, the details of these processes are still not understood and in some cases remain controversial. Here, we aim to elucidate features of the FEN1 protein and substrates required for global DNA bending and local DNA unpairing (*i.e.* transfer to the active site). We also investigate the relationship of these processes to 5′-flap accommodation and explore the orientation of the 5′-portion of substrates that is not visible in current x-ray structures. Our combined results describe substrate and protein requirements for DNA bending and unpairing, and in turn Okazaki fragment processing, providing important insights into the FEN1 catalytic cycle.

## Experimental Procedures

### 

#### 

##### DNA Constructs

The oligonucleotide sequences are given in Table 1. DNA oligonucleotides including those containing 5′-FAM, 5′-biotin, internal TAMRA and fluorescein, and 2-aminopurine (2AP) substitutions were purchased with HPLC purification from DNA Technology A/S. The phosphoramidite synthons used for 5′-FAM, 5′-biotin, internal TAMRA dT, and internal fluorescein dT modifications were 6-carboxyfluorescein-aminohexyl amidite, *N*-DMT-biotinyl-2-aminoethoxyethanol amidite, 5′-DMT-T(TEG-TAMRA), and fluorescein T amidite, respectively, and were purchased from Biosearch Technologies Inc. 2AP was incorporated using 5′-(4,4′-dimethoxytrityl)-*N*^2^-(dimethylformamidine)-2′-deoxypurine riboside-3′-[(2-cyanoethyl)-(*N*,*N*-diisopropyl)]phosphoramidite obtained from Link Technologies Ltd. DNA concentrations were determined by UV absorbance at 260 nm (20 °C) using extinction coefficients generated by the Integrated DNA Technologies oligo analyzer 3.1 tool.

Substrate constructs are summarized in Table 2. FRET substrates were designed by modeling a range of different fluorophore positions using the accessible volume approach (18) on both duplex and bent hFEN1 substrate DNAs (obtained by extending the existing DNA helixes in the crystal structure of hFEN1-DNA (8)). Labeling sites were chosen to maximize the FRET change upon bending. FRET substrates (Table 2) were assembled by heating the appropriate 3′-flap, 5′-flap/exo, and template strands in 1:1.1:1 ratio in 50 mm HEPES, pH 7.5, and 100 mm KCl to 80 °C for 5 min and then cooling to room temperature. For comparison, a DNA duplex was also created as above with Tcdonor (see Table 1) and template strands in a 1:1 ratio. 2AP constructs and the kinetic substrate KDF were formed by heating the appropriate exo/5′-flap strands with the complementary template in a 1:1.1 ratio at 80 °C for 5 min in 50 mm Tris-HCl, pH 7.5, and 100 mm KCl with subsequent cooling to room temperature.

##### Enzymes

hFEN1 and mutants were overexpressed and purified as described (8, 13).

##### Florescence Resonance Energy Transfer

FRET efficiencies (*E*) were determined using the (ratio)_A_ method (19) by measuring the enhanced acceptor fluorescence at 37 °C. The steady state fluorescent spectra of 10 nm nonlabeled (NL) trimolecular, donor-only labeled (DOL), and doubly labeled (DAL) DNA substrates (Table 2) were recorded using a Horiba Jobin Yvon FluoroMax-3® fluorometer. For direct excitation of the donor (fluorescein, DOL) or acceptor (TAMRA, AOL), the sample was excited at 490 or 560 nm (2-nm slit width), and the emission signal was collected from 515–650 or 575–650 nm (5-nm slit width). Emission spectra were corrected for buffer and enzyme background signal by subtracting the signal from the nonlabeled (NL) DNA sample. In addition to 10 nm of the appropriate DNA construct, samples contained 10 mm CaCl_2_ or 2 mm EDTA, 110 mm KCl, 55 mm HEPES, pH 7.5, 0.1 mg/ml bovine serum albumin, and 1 mm DTT. The first measurement was taken prior to the addition of protein with subsequent readings taken on the cumulative addition of the appropriate enzyme in the same buffer, with corrections made for dilution. Transfer efficiencies (*E*) were determined according to Equations 1–3, where *F*_DA_ and *F*_D_ represent the fluorescent signal of the doubly labeled DNA (DAL) and donor-only labeled DNA (DOL) at the given wavelengths, respectively (*e.g.* F_DA_(λ_EX_^D^, λ_EM_^A^) denotes the measured fluorescence of acceptor emission upon excitation of the donor, for DAL DNA); ϵ^D^ and ϵ^A^ are the molar absorption coefficients of donor and acceptor at the given wavelengths; and ϵ^D^(490)/ϵ^A^(560) and ϵ^A^(490)/ϵ^A^(560) are determined experimentally from the absorbance spectra of doubly labeled molecules (DAL) and the excitation spectra of singly TAMRA-only labeled molecules (AOL), respectively. Energy transfer efficiency (*E*) was fitted by nonlinear regression in the Kaleidagraph program to Equation 4, where *E*_max_ and *E*_min_ are the maximum and minimum energy transfer values, [S] is the substrate concentration, [P] is the protein concentration, and *K*_bend_ is the bending equilibrium dissociation constant of the protein substrate [PS] complex. All experiments were repeated in triplicate.

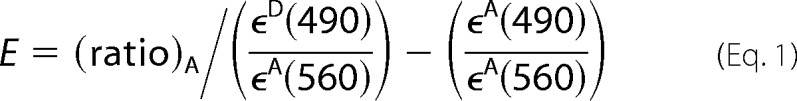
 where

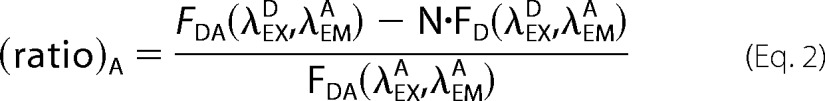
 and

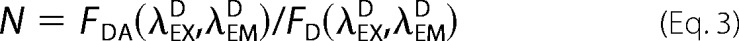


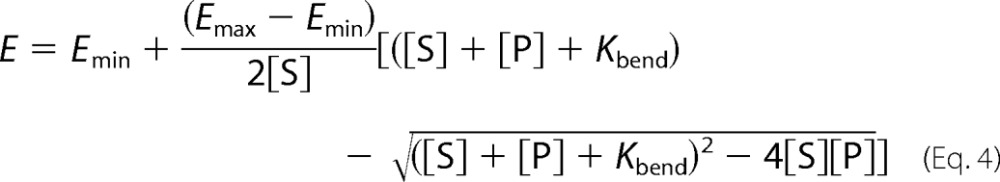
 Donor (fluorescein) was excited at 490 nm with emission sampled as the average value of the signal between 515 and 525 nm, and acceptor (TAMRA) was excited at 560 nm with emission averaged between 580 and 590 nm. For FRET experiments involving substrate bound to streptavidin, 5 molar equivalents of streptavidin were preincubated with the biotinylated substrate in buffer containing 10 mm CaCl_2_, 55 mm HEPES, pH 7.5, 110 mm KCl, 1 mg/ml BSA, and 1 mm DTT for 10 min at room temperature before proceeding as above.

##### Determination of the Maximal Single Turnover Rate of Reaction (k_STmax)_

Maximal single turnover rates of reaction were determined using the KDF substrate (Table 2) and rapid quench apparatus (for WT-hFEN1 and Y40A) or manual sampling (for D181A) at 37 °C and pH 7.5, as described (20).

##### CD Spectroscopy

Samples containing 10 μm of the appropriate (2AP)_2_ DNA construct (Table 2), 50 mm Tris-HCl, pH 7.5, 100 mm KCl, 1 mm DTT and, where appropriate, 12.5 μm protein and either 10 mm CaCl_2_ or 10 mm CaCl_2_ + 25 mm EDTA were prepared with subsequent acquisition of CD spectra (300–480 nm) at 20 °C using a JASCO J-810 CD spectrophotometer as described in detail (17). The CD spectra were plotted as Δϵ per mol 2AP residue *versus* wavelength. Each measurement was independently repeated typically in triplicate.

## Results

### Global DNA Conformational Change

#### 

##### Substrate Design for DNA Bending

**FIGURE 2.**
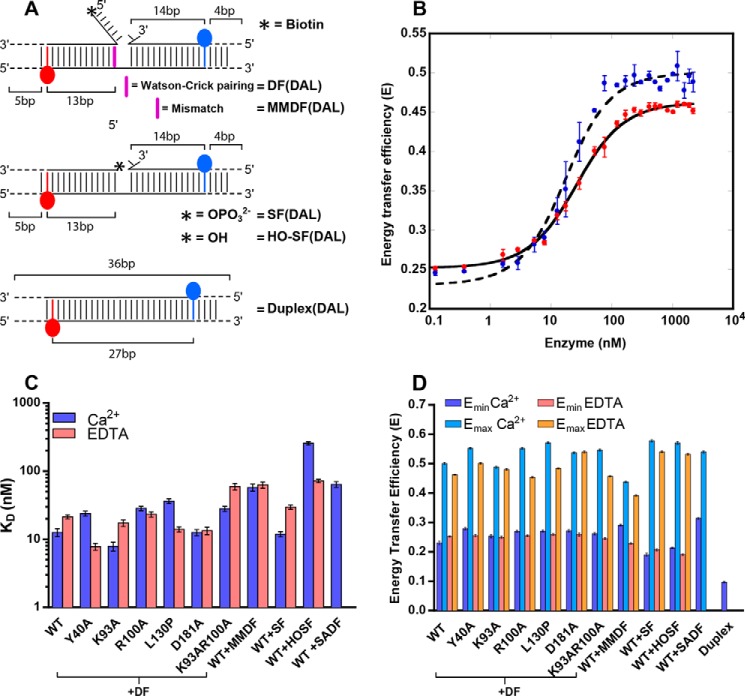
**FRET data showing DNA bending on complexation with hFEN1 and mutants.** *A*, schematic of double flap (DF, endonucleolytic) and single flap (SF, exonucleolytic) DNA constructs (Table 2) used in FRET studies, donor = fluorescein (*blue*) and acceptor = TAMRA (*red*). Nonlabeled (NL), donor only (DOL), acceptor-only (AOL), and donor and acceptor (DAL) versions of these constructs were used. *B*, variation in energy transfer efficiency of DF(DAL) upon addition of WT hFEN1 measured at pH 7.5 and 37 °C in the presence of Ca^2+^ ions (*blue*) or EDTA (*red*) fitted to Equation 4. *C*, derived (Equation 4) values of *K*_bend_ for the DF (double flap) and SF (single flap) substrates (Table 2) with WT and mutated hFEN1s as indicated in Ca^2+^ (*purple*) and EDTA (*pink*). MMDF contained a +1 mismatch, 5′-hydroxyl single (3′) flap (*HOSF*) lacked a 5′-phosphate, and SADF had a 5′-conjugated streptavidin. Standard errors from repeat experiments are shown. *D*, derived (Equation 4) minimum (*E*_min_) and maximum (*E*_max_) energy transfer in Ca^2+^ (*blue*) and EDTA (*orange*) corresponding to the indicated protein with DF (double flap) or SF (single flap) substrates as in *C*. Duplex DNA was measured for comparison without protein in Ca^2+^-containing buffer. Standard errors from repeat experiments are shown.

To study global conformational change of DNA substrates (Fig. 1*B*), we used FRET to detect duplex-duplex bending upon binding to human FEN1 (hFEN1) (Fig. 2) (7, 9, 10). Donor- and acceptor-labeled (DAL) substrates were assembled from three oligonucleotides, a TAMRA-labeled template strand, a fluorescein-labeled 3′-flap strand and an unlabeled 5′-flap/exo strand (Tables 1 and 2 and Fig. 2*A*). The positions of the fluorophores were chosen to maximize the FRET change observed upon substrate bending. In addition, donor only labeled (DOL), acceptor-only labeled (AOL), and nonlabeled (NL) versions of the substrates were also prepared (Table 2) to determine FRET efficiencies using the (ratio)_A_ method (19). In double flap (DF) FRET substrates, the 5′-flap strand carried a terminal 5′-biotin to facilitate experiments with streptavidin; this label did not affect FRET behavior (data not shown). To reduce any ambiguity in interpretation of our results, all substrates used in our studies were designed to be static (*i.e.* the flaps were noncomplementary to the template strand). Such static flaps permit clearer interpretation of experimental data but are known to behave identically to their equilibrating counterparts in hFEN1 reactions (6). For comparison, we also created the equivalent DAL duplex to the flapped DNAs (Table 2 and Fig. 2*A*).

**TABLE 1:**
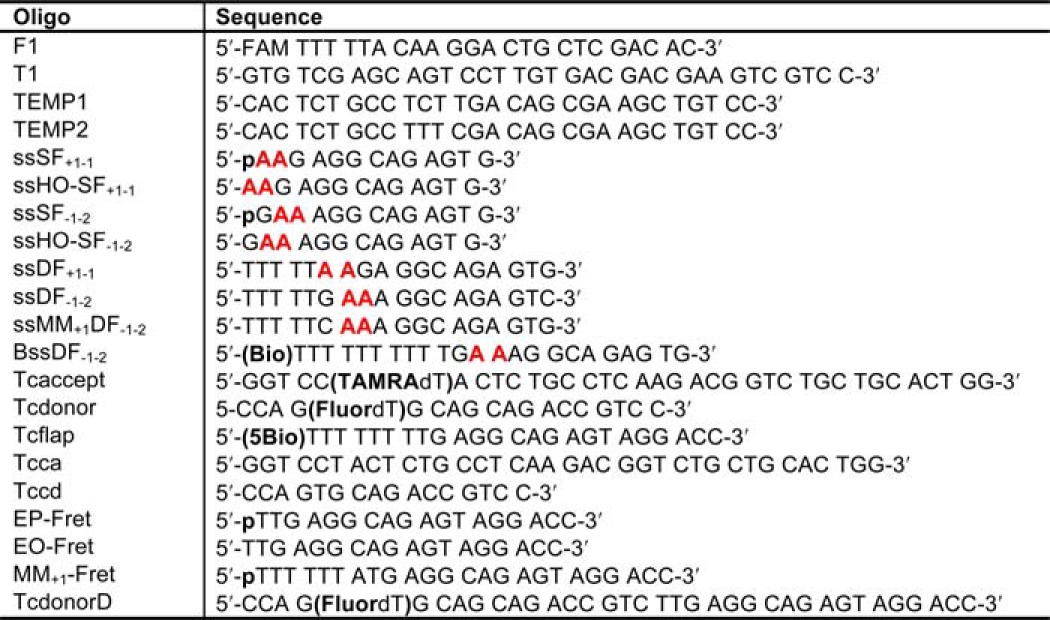
**Sequences of oligonucleotides used to construct substrates for FRET, kinetic, and ECCD experiments** A (in red), 2-aminopurine; **Bio**, biotin; **TAMRA**, tetramethylrhodamine; **Fluor**, internal fluorescein; **FAM**, fluorescein; **p**, phosphate.

**TABLE 2:** **Oligonucleotide combinations used to make the substrate constructs for FRET, kinetic, and ECCD experiments**

Construct	Composition
KDF	F1 + T1
SF_+1−1_	TEMP1 + ssSF_+1–1_
HO-SF_+1−1_	TEMP1 + ssHO-SF_+1–1_
DF_+1−1_	TEMP1 + ssDF_+1–1_
BDF_−1−2_	TEMP2 + BssDF_−1–2_
SF_−1−2_	TEMP2 + ssSF_−1–2_
HO-SF_−1−2_	TEMP2 + ssHO-SF_−1–2_
MM_+1_DF_−1−2_	TEMP2 + ssMM_+1_DF_−1–2_
DF (NL)	Tcflap + Tcca + Tccd
DF (DOL)	Tcflap + Tcca + Tcdonor
DF (AOL)	Tcflap + Tccd + Tcaccept
DF (DAL)	Tcflap + Tcdonor + Tcaccept
SF (NL)	EP-Fret + Tcca + Tccd
SF (DOL)	EP-Fret + Tcca + Tcdonor
SF (AOL)	EP-Fret + Tccd + Tcaccept
SF (DAL)	EP-Fret + Tcdonor + Tcaccept
HO-SF (NL)	EO-Fret + Tcca + Tccd
HO-SF (DOL)	EO-Fret + Tcca + Tcdonor
HO-SF (AOL)	EO-Fret + Tccd + Tcaccept
HO-SF (DAL)	EO-Fret + Tcdonor + Tcaccept
MM_+1_-DF (NL)	MM_+1_-Fret + Tcca + Tccd
MM_+1_-DF (DOL)	MM_+1_-Fret + Tcca + Tcdonor
MM_+1_-DF (AOL)	MM_+1_-Fret + Tccd + Tcaccept
MM-DF (DAL)	MM-Fret + Tcdonor + Tcaccept
Duplex (DOL)	TcdonorD + Tcca
Duplex (DAL)	TcdonorD + Tcaccept

To prevent cleavage of the substrate, all experiments were carried out in the absence of the viable cofactor Mg^2+^. Because divalent metal ions are required for accommodation of the 5′-flap and for DNA conformational changes that lead to reaction (12, 17), we carried out experiments with or without catalytically nonviable Ca^2+^ ions, allowing us to investigate the relationship between DNA bending and other events of the hFEN1 catalytic cycle. Calcium ions are competitive inhibitors of Mg^2+^-supported 5′-nuclease reactions, implying they occupy similar sites in the protein (15, 22); they have also been shown to facilitate 5′-flap threading and local DNA conformational changes (12, 17). Analysis of samples after both FRET and later CD experiments demonstrated negligible extent of reaction under all the conditions used (data not shown).

##### Catalytically Important Active Site Features Are Not Required for DNA Junction Bending

The FRET efficiency of DF(DAL) alone was similar ±Ca^2+^ (0.23–0.25) but was significantly greater than the corresponding duplex (0.1) (Fig. 2*D*). This indicates that the DF substrate has an overall conformation that is more bent than duplex DNA, even before the addition of protein. This is in line with single molecule observations where a double flap was seen to sample both a linear stacked and a bent conformation (9). Sequential addition of WT hFEN1 to DF(DAL) produced an increase in corrected FRET signal until a plateau was reached at saturating protein, regardless of whether divalent ions were present or not (Fig. 2, *B* and *D*). When DF (DAL) was fully bound to hFEN1 (FRET efficiency at end point), a slightly higher energy transfer value was reproducibly observed with Ca^2+^ ions present (Fig. 2, *B* and *D*). The origin of this end point difference is unknown. Nevertheless, the derived equilibrium dissociation constants *K*_bend_ ± Ca^2+^ only varied by a factor of two (13 ± 1.7 nm with Ca^2+^, 21 ± 1.4 nm without), implying that the presence of divalent ions is not required for DNA to adopt a bent conformation when bound to hFEN1 (Fig. 2, *B* and *C*). Because divalent ions are required for the threading of 5′-flaps (12) and the transfer of the scissile phosphodiester to the active site (17), these results suggest that the DF substrate binds with similar affinity regardless of whether either of these conformational changes have taken place. This is consistent with the crystal structure that shows that most of the interaction surface area is with the duplex portions of the substrate (8).

To investigate the requirements for bending of DF DNA, we also tested mutated hFEN1s K93A, R100A, K93A/R100A, L130P, Y40A, and D181A (Fig. 1*D*). Superfamily conserved residues Lys^93^ and Arg^100^ are located at the base of α4 forming part of the hFEN1 helical gateway (8) from where they protrude into the hFEN1 active site and are not predicted to be involved in substrate interactions until the DNA is positioned to react. Leu^130^ is a component of the helical cap (α5) and is removed from the active site, although the mutation L130P is presumed to interfere with formation of the secondary structure of the cap (13). Tyr^40^ is an α2 gateway residue seen to interact with the +1 nucleobase (numbered relative to the scissile phosphate diester; Fig. 1*C*) of the DNA substrate when base-paired (8), whereas it stacks on the −1 nucleobase after reaction as seen in hFEN1 product structures (Fig. 1*D*). Asp^181^ is an active site carboxylate in direct contact with the catalytic metal ions in hFEN1 structures (8). Mutation of Asp^181^ may alter the number of metal ions bound and/or their precise positioning. Earlier studies have shown that under maximal single turnover conditions, the mutations K93A, R100A, K93A/R100A, and L130P decrease the rate of the hFEN1 reaction by factors of at least 2,000 (12, 13). To determine the effects of the Y40A and D181A mutations, we measured the maximal single turnover rate constants (*k*_STmax_) using KDF substrate (Tables 1 and 2) and compared them with the WT protein (*k*_STmax_ = 740 min^−1^) (data not shown). For Y40A, *k*_STmax_ = 7.91 ± 0.01 min^−1^, and for D181A, *k*_STmax_ = 0.075 ± 0.003 min^−1^, corresponding to rate decreases of 10^2^ and 10^4^, respectively. Thus, all the mutations studied have substantive and in most cases, very severe impacts on hFEN1 catalysis.

DF (DAL) adopted a bent conformation when bound to all the mutated proteins as seen by an increase in FRET signal upon addition of hFEN1. As with the wild type protein, only subtle variations in *K*_bend_ were observed with and without divalent metal ions (2-fold at most) (Fig. 2*C*). The exception was Y40A, where mutation stabilized the hFEN1-DNA complex in the presence of EDTA. Only small changes in *K*_bend_ were observed relative to the WT protein ±Ca^2+^ (less than 3-fold at most), indicating that none of the mutated residues are critical to DNA binding and bending. Like the WT protein, differences between the FRET efficiency at the end point ±Ca^2+^ were also observed with Y40A, R100A, K93A/R100A, and L130P with titrations in Ca^2+^ buffer producing a higher value (Fig. 2*D*). In contrast, the end points with D181A and K93A remained constant ±Ca^2+^. Notably, all the altered FEN1 proteins have *K*_bend_ values in the low nanomolar range ± Ca^2+^, demonstrating that they will all fully bind substrate under the conditions of the local DNA unpairing (2AP)_2_ CD experiments described later (12.5 μm protein, 10 μm DNA).

##### A Mismatch at the +1 Position of the Substrate Does Not Prevent Bending

Previously, we showed that double-flap substrates bearing a mismatch at the +1 position (numbering relative to scissile phosphodiester bond in the 5′-flap/exo strand; Fig. 1*C*) produced reduced reaction rates and reduced reaction-site specificity (16). This shows that the DNA base pair integrity at the +1 position is a requirement for optimal hFEN1 reaction. To determine whether a mismatch at +1 affects the ability to bind and bend substrate DNA, we prepared the appropriate construct MMDF(DAL) (Fig. 2*A*) and performed the same FRET measurements (Fig. 2*C*). Like the alteration of conserved active site residues, the presence of a mismatch at the +1 position does not prevent bending, but it does weaken substrate affinity 4–5-fold (in Ca^2+^ DF *K*_bend_ = 13 ± 1.7 nm, MMDF *K*_bend_ = 58 ± 6.8 nm).

##### A 5′-Flap Is Not Required for DNA Bending

An initial conundrum in the reactions of 5′-nucleases concerned their ability to carry out both endonucleolytic reactions on substrates that possessed 5′-flaps and 5′-exonucleolytic reactions on substrates that lacked such flaps. To test whether the absence of 5′-flap altered the stability of hFEN1-DNA complexes, we carried out a FRET experiment with a single flap substrate (SF(DAL)) that lacked the 5′-flap (Fig. 2*A*). Consistent with the crystal structure and the fact that hFEN1 reaction is susceptible to dsDNA (nicked) product inhibition (8, 20), the absence of a 5′-flap did not significantly alter the stability of the complex or the ability to bend (*K*_bend_ = 12 ± 1.1 nm with Ca^2+^, 20 ± 2.1 nm without) (Fig. 2*C*). This is also consistent with similar *K_m_* values observed earlier for exonucleolytic substrates bearing a 3′-flap compared with double flaps (20). However, the dissociation constant of SF substrate was sensitive to the status of the 5′-terminus. HO-SF (DAL), which lacked a 5′-phosphate monoester, was bound an order of magnitude more weakly by the protein in the presence of Ca^2+^ ions, and binding was also altered in EDTA to a lesser extent (Fig. 2*C*). This suggests that the 5′-phosphate forms an interaction with the protein facilitated by the local DNA conformational changes that occur in the presence of Ca^2+^ ions. Nevertheless, even HO-SF(DAL) would be fully bound to the protein under the conditions used to probe local DNA conformational changes by CD below. Like DF(DAL), SF(DAL) and HO-SF(DAL) also had a greater FRET value in the absence of protein (0.19–0.21) than the corresponding duplex (0.1), suggesting that the SF substrates can adopt a bent conformation in the absence of protein (Fig. 2*D*).

##### Accommodation of the 5′-Flap Is Not Required for DNA Bending

Although FEN1 substrates correctly positioned to react have yet to be observed crystallographically, it is suggested that the 5′-flap departs from the active site passing underneath the helical cap through the hole created by the cap (top of α4 and α5) and gateway (base of α4 and α2) (Fig. 1, *B* and *D*) (1, 8, 12–14). Evidence for this so-called threading hypothesis came from experiments where streptavidin is added to 5′-biotin-labeled substrates before or after binding to the protein (12, 14). Prior conjugation—assumed to “block” substrate threading—severely retards FEN1 action, but conjugation to preformed DNA-protein complex does not affect the reaction rate. Furthermore, only this latter “trapped” substrate cannot exchange with competitor DNA.

We wished to ascertain whether, when present, accommodation of the 5′-flap is necessary for global substrate bending. A 5′-strepavidin complex with DF(DAL) (12) was used (blocked SADF) and showed a higher FRET efficiency in the absence of protein (Fig. 2*D*). This suggests a more bent overall conformation than uncomplexed DNA, likely because of the presence of a bulky streptavidin homotetramer conjugated to the 5′-terminus. Nevertheless, the blocked SADF with hFEN1-Ca^2+^ had a similar FRET efficiency at end point as the unmodified substrate, albeit with a 5-fold increase in *K*_bend_ (Fig. 2*C*). This result demonstrates that accommodation of the 5′-flap underneath the helical cap is not required for global substrate bending.

### Local DNA Conformational Change of the Reacting Duplex

#### 

##### A Substrate 5′-Flap Is Not Required for Local DNA Conformational Change

In hFEN1-product structures, the −1 nt is unpaired and extrahelical (Fig. 1, *B* and *D*) such that its 5′-phosphate monoester contacts active site metal ions, whereas the adjacent −2 nt remains base-paired (8) (numbering of 5′-flap/exo strand, (Fig. 1*C*)). In contrast, structures of hFEN1-substrate DNA, where the substrate has no 5′-phosphate monoester, showed a base-paired substrate close to but not in the active site. Thus, it was deduced that 2 nt of the substrate unpair to allow the scissile phosphate to contact active site ions. We previously studied this local DNA conformational change using substrate or product constructs labeled with tandem 2APs at the −1 and −2 positions (DF_−1−2_ and P_−1−2_, respectively) (17). An exciton coupling between the adjacent 2APs produces a signal in the low energy region of the CD spectrum, the magnitude of which varies depending upon the relative orientation of the electronic transition dipole moments of the nucleobases. This exciton-coupled CD (ECCD) signal is readily followed because it is partially visible in a region of the spectrum where unmodified DNA bases and protein are transparent (24, 25). When either DF_−1−2_ or P_−1−2_ was bound to hFEN1 in EDTA buffer, a strong ECCD signal was observed (λ_max_ = 326 nm), consistent with the 2APs remaining stacked in the duplex. In the presence of hFEN1-Ca^2+^, the signal was dramatically reduced to nearly zero. This was deduced to reflect the DNAs adopting a conformation of the kind seen in the product crystal structure, with transfer of the 5′-nucleotide of product, or the +1 and −1 nt of substrate, to the active site (Fig. 1*D*).

**FIGURE 3.**
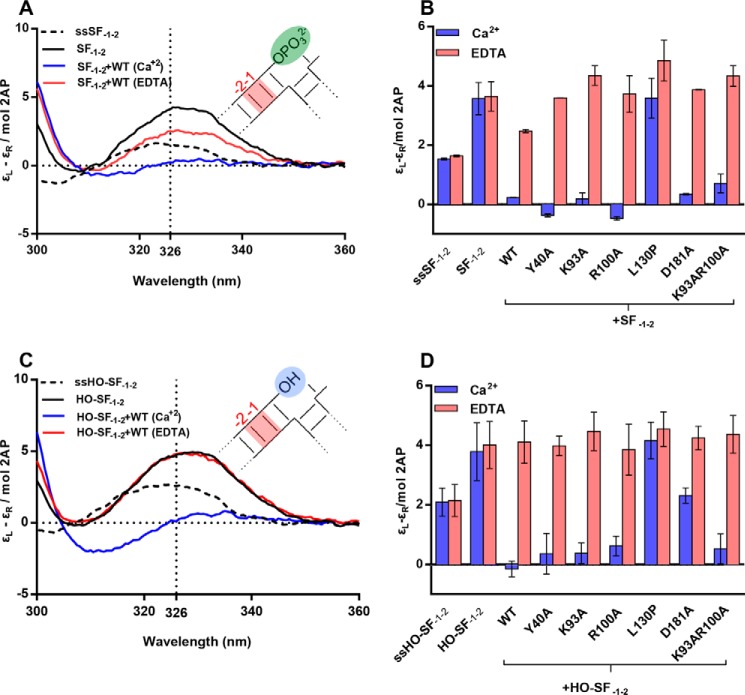
**hFEN1 and mutant mediated conformational change of 2AP-containing single flap SF_−1−2_ monitored by ECCD.** All measurements were carried out at 20 °C and pH 7.5. *A*, divalent metal ion-dependent reduction in 2AP exciton coupling signal occurred when substrate SF_−1−2_ was bound to hFEN1, indicative of local substrate conformational change. Unbound SF_−1−2_ (*black*), the corresponding single strand (ssSF_−1−2_, *dashed line*) and SF_−1−2_ bound to hFEN1 (*blue*) all in Ca^2+^-containing buffer. SF_−1−2_ bound to hFEN1 in buffer containing 25 mm EDTA (*red*). *B*, comparison of molar ellipticity per 2AP residue at 326 nm of SF_−1−2_ bound to WT- and mutant hFEN1s in Ca^2+^ (*purple*) and EDTA (*pink*) buffers. Standard errors from repeat experiments are shown. *C*, divalent metal ion-dependent reduction in 2AP exciton coupling signal occurred when substrate HO-SF_−1−2_, which lacks a 5′-phosphate, was bound to hFEN1, indicative of local substrate conformational change. Unbound HO-SF_−1−2_ (*black*), the corresponding single strand (ssHO-SF_−1−2_, *dashed line*) and HO-SF_−1−2_ bound to hFEN1 (*blue*) all in Ca^2+^-containing buffer. HO-SF_−1−2_ bound to hFEN1 in buffer containing 25 mm EDTA (*red*). *D*, comparison of molar ellipticity per 2AP residue at 326 nm of single flap HO-SF_−1−2_ free or bound to WT and mutant hFEN1s in Ca^2+^ (*purple*) and EDTA (*pink*) buffers. The unbound corresponding single strand is also shown.

By analogy to these earlier experiments, 2APs were located at the −1 and −2 positions of a SF substrate (SF_−1−2_) to test whether exonucleolytic substrates lacking the 5′-flap were also unpaired by hFEN1-Ca^2+^ (Tables 1 and 2 and Fig. 3). As seen previously, the ECCD signal of the isolated (2AP)_2_ single-strand (ssSF_−1−2_) was increased in magnitude, and the maximum was red-shifted to 326 nm upon forming the duplex SF_−1−2_ (Fig. 3*A*) (17). On addition of hFEN1-Ca^2+^ to this substrate, the signal was dramatically reduced to near zero (Fig. 3, *A* and *B*). This behavior is similar to that observed earlier with DF_−1−2_ or P_−1−2_ (17). When EDTA was added to the hFEN1-Ca^2+^·SF_−1−2_ sample, a strong ECCD signal at 326 nm was restored. This demonstrates that the 5′-flap is not required for a change in respective orientation of the −1 and −2 nt, while confirming the presence of active site divalent metal ion(s) is essential. Moreover, both exonucleolytic (SF) and endonucleolytic (DF) substrates undergo analogous local DNA conformational changes.

##### FEN1 Conserved Residues Are Not Required for −1−2 Local DNA Conformational Change

Similar experiments were conducted with SF_−1−2_ and mutant hFEN1 proteins. Fig. 3*B* shows the magnitude of the ECCD signal at 326 nm for each mutated protein ±Ca^2+^. K93A, R100A, Y40A, and K93A/R100A were all capable of effecting local conformational change of SF_−1−2_ in the presence of Ca^2+^, with K93A most closely matching the spectra obtained with WT protein in Ca^2+^. As seen previously with DF_−1−2_ (13, 17), spectra of SF_−1−2_ produced by R100A, Y40A, and K93A/R100A with Ca^2+^ contained an additional minimum at 310 nm (data not shown). This suggests an altered orientation of the −1 and −2 nt to that produced by WT and K93A hFEN1s. We found that D181A-Ca^2+^ was able to bring about an analogous conformational change to WT protein (Fig. 3*B*), which was surprising given that no active site metal ions were visible in an x-ray structure of D181A bound to SF DNA substrate in the presence of Ca^2+^ and the DNA remained base-paired (8). In contrast, the ECCD signal at 326 nm with L130P was similar ±Ca^2+^, indicating that this protein does not facilitate the local DNA conformational change. Together, these results demonstrate that conserved residues are not required to bring about a change in the orientation of the −1 and −2 nt in exonucleolytic DNA substrates, although the intact secondary structure of the helical cap is. The results obtained with mutated hFEN1s strongly resemble those previously obtained with DFs (13, 17), underscoring that there are no overall differences between the behaviors of exonucleolytic (without 5′-flap) and endonucleolytic (with 5′-flap) hFEN1 substrates.

##### A 5′-Phosphate Is Not Required for Local DNA Conformational Change Monitored at the −1 and −2 nt

In the exonucleolytic FEN1 substrate SF_−1−2_, the +1 nt has a terminal 5′-phosphate, whereas the double flap substrate DF_−1−2_ has a 5′-phosphate diester (followed by the flap) in the corresponding position. Both substrates underwent a similar local DNA conformational change when bound by hFEN1-Ca^2+^. A SF substrate lacking a 5′-phosphate (*i.e.* 5′-OH) crystallized with hFEN1 in base-paired form, despite the presence of active site metal ions (8). Furthermore, we previously reported that SF substrates lacking the 5′-phosphate monoester showed a 20-fold decrease in reaction efficiency, and we hypothesized that this was due to the inability to affect the local conformational change. To test whether the 5′-phosphate monoester is required for reorientation of the −1 and −2 nt, we created a substrate lacking the 5′-phosphate, HO-SF_−1−2_. Surprisingly, we observed that this substrate underwent a change in orientation of the 2APs upon addition of hFEN1-Ca^2+^ with the signal reducing close to zero at 326 nm (Fig. 3*C*). However, unlike the 5′-phosphorylated SF_−1−2_, the spectra also contained a minimum at 315 nm. Thus, the presence of a 5′-phosphate is not required for the WT protein to bring about local DNA conformational change involving the −1 and −2 nt in substrate DNAs, but the orientation of the 2APs may differ from that adopted by the 5′-phosphorylated form (Fig. 3, *A* and *C*). Additionally, all the mutated proteins had the same response to HO-SF_−1−2_ asSF_−1−2_ with the exception of D181A, where hFEN1-Ca^2+^ reduced the ECCD signal to a lesser extent (Fig. 3*D*).

##### Streptavidin Blocking of 5′-Flaps Prevents Local DNA Conformational Change

**FIGURE 4.**
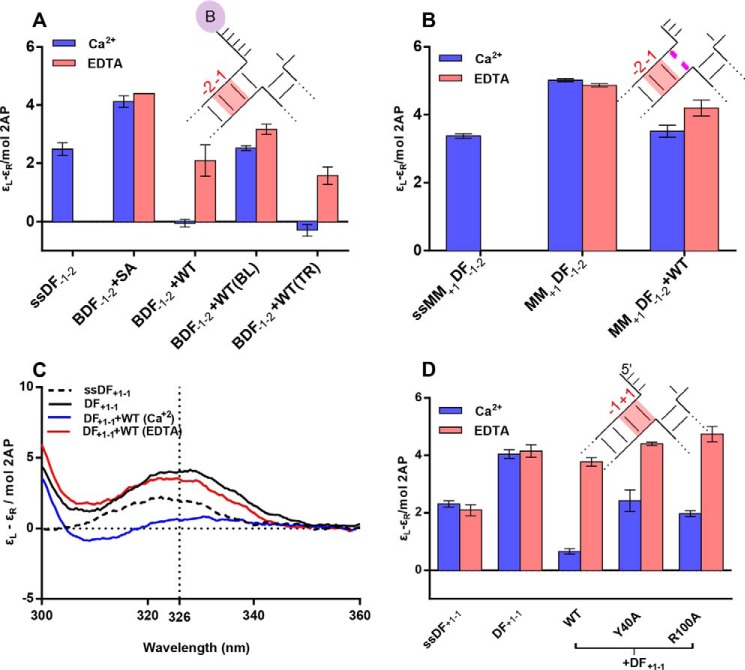
**ECCD monitored conformational change of +1−1 2AP and 5′-modified −1−2 double flap substrates.** All measurements were carried out at 20 °C and pH 7.5. *ss*, single strand. Standard errors from repeat experiments are shown. *A*, comparison of molar ellipticity per 2AP residue at 326 nm of 5′-streptavidin blocked (*BL*) and free and bound to hFEN1 and streptavidin-trapped (*TR*) complexes in Ca^2+^ (*purple*) and EDTA (*pink*) buffers. Blocked complex was formed by adding streptavidin to the substrate before the addition of hFEN1, whereas trapped was formed by adding streptavidin to the preformed hFEN1-Ca^2+^-BDF complex. *B*, comparison of molar ellipticity per 2AP residue at 326 nm of a doubled flap substrate with a +1 mismatch (MMDF_−1−2_) when free and bound to WT-hFEN1 in Ca^2+^ (*purple*) and EDTA (*pink*) buffers. The corresponding single strand is also shown. *C*, divalent metal ion-dependent reduction in 2AP exciton coupling signal occurred when substrate DF_+1−1_ was bound to hFEN1, indicative of local substrate conformational change. Unbound DF_+1−1_ (*black*), the corresponding single strand (ssDF_+1−1_, *dashed line*) and DF_+1−1_ bound to hFEN1 (*blue*) all in Ca^2+^-containing buffer. DF_+1−1_ bound to hFEN1 in buffer containing 25 mm EDTA (*red*). *D*, comparison of molar ellipticity per 2AP residue of double flap DF_+1−1_ at 326 nm when free and bound to WT and R100A hFEN1s in Ca^2+^ (*purple*) and EDTA (*pink*) buffers. The corresponding single strand is also shown. Standard errors from repeat experiments are shown.

To test whether the severely reduced reaction rates observed with 5′-streptavidin blocked substrates resulted from inability to transfer substrate to the active site, we created a 5′-biotinylated double flap with 2AP at −1 and −2, BDF_−1−2_. The addition of biotin did not alter behavior of the substrate in ECCD experiments (Fig. 4*A*), but its behavior when the 5′-flap was blocked with streptavidin was markedly different. In this case, addition of hFEN1-Ca^2+^ did not alter the ECCD signal, indicating that local substrate conformational change is prevented by the addition of the streptavidin block. In contrast, when streptavidin was added to trap a preformed complex of hFEN1-Ca^2+^·BDF_−1−2_, the ability to change the conformation of the substrate was retained. These results demonstrate that proper accommodation of the 5′-flap of the DNA substrate is required for the local conformational change necessary for reaction.

##### A Watson-Crick Base Pair Is Required at the Terminus of the Hydrolyzed Duplex

To test whether the decreased rate and specificity with mismatched substrates could be attributed to inhibition of the local DNA conformational change, we created a double flap substrate with a +1 C-C mismatch retaining 2APs at positions −1 and −2, denoted MM_+1_DF_−1−2_ (Fig. 4*B*). The ECCD signal produced by WT hFEN1-Ca^2+^ and MM_+1_DF_−1−2_ was decreased slightly compared with that for the mismatch substrate alone or the same sample in EDTA but did not approach the nearly zero signal produced with fully base-paired substrate under these conditions. This implies that although the local DNA structure of the mismatched substrate may be subtly altered by hFEN1-Ca^2+^, it does not adopt the same conformation as the Watson-Crick base-paired substrate, or there is a significant change in the partition between the base-paired and active site positioned forms.

##### Local DNA Conformational Change at the +1−1 Position Requires Conserved Residues and a +1 Phosphate

Placing the scissile phosphodiester bond on hFEN1 active site metal ions is presumed to require that both the +1 and −1 nt of the substrate unpair from duplex (Fig. 1*C*). Because there are currently no x-ray structures of hFEN1 in complex with substrate positioned to react, the relative juxtaposition of the −1 and +1 nucleobases in this catalytically competent state are unknown. To use ECCD to inform on this state, we created single flap SF_+1−1_ and double flap DF_+1−1_ substrates containing tandem 2APs at the −1 and +1 positions (Fig. 4, *C* and *D*, and 5, *A* and *B*). In both cases, addition of Ca^2+^ to hFEN1 complexes with the respective substrates substantially decreased the ECCD signal at 326 nm. This implies that in the presence of hFEN1-Ca^2+^ stacking interactions between the +1 and −1 nt are significantly altered.

**FIGURE 5.**
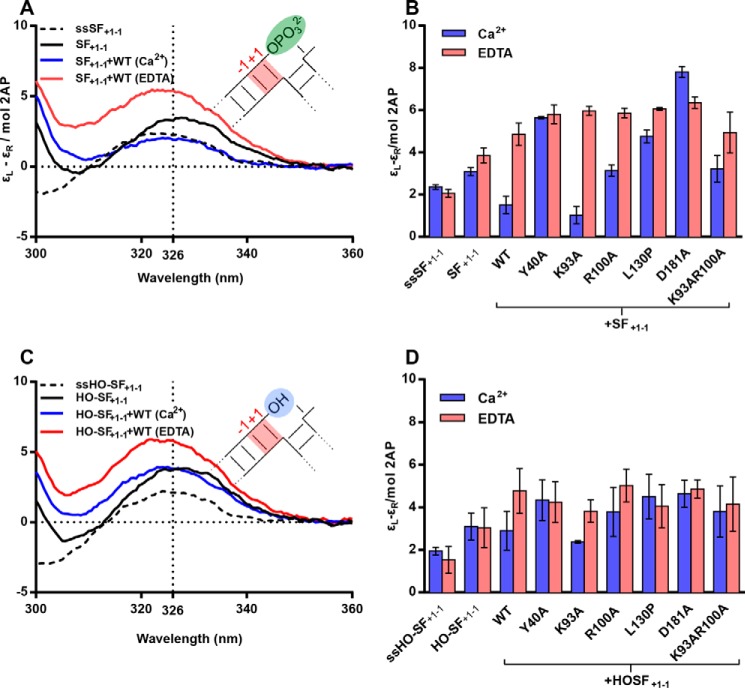
**ECCD monitored conformational change of single flap +1−1 2AP containing substrates upon binding hFEN1 and mutants.** All measurements were carried out at 20 °C and pH 7.5. *ss*, single strand. *A*, divalent metal ion-dependent reduction in 2AP exciton coupling signal occurred when single flap SF_+1−1_ was bound to hFEN1, indicative of local substrate conformational change. Unbound SF_+1−1_ (*black*), the corresponding single strand (ssSF_+1−1_, *dashed line*) and SF_+1−1_ bound to hFEN1 (*blue*) all in Ca^2+^-containing buffer. SF_+1−1_ bound to hFEN1 in buffer containing 25 mm EDTA (*red*). *B*, comparison of molar ellipticity per 2AP residue of SF_+1−1_ at 326 nm when free and bound to WT and mutant hFEN1s in Ca^2+^ (*purple*) and EDTA (*pink*) buffers. The corresponding single strand is also shown. Standard errors from repeat experiments are shown. *C*, a small divalent metal ion-dependent reduction in 2AP exciton coupling signal occurred when single flap HO-SF_+1−1_ that lacks a 5′-phosphate was bound to hFEN1, indicative of deficiency in bringing about local substrate conformational change. Unbound HO-SF_+1−1_ (*black*), the corresponding single strand (ssHO-SF_+1−1_, *dashed line*) and HO-SF_+1−1_ bound to hFEN1 (*blue*) all in Ca^2+^-containing buffer. HO-SF_+1−1_ bound to hFEN1 in buffer containing 25 mm EDTA (*red*). *D*, comparison of molar ellipticity per 2AP residue of single flap HO-SF_+1−1_ at 326 nm when free and bound to WT and mutant hFEN1s in Ca^2+^ (*purple*) and EDTA (*pink*) buffers. The corresponding single strand is also shown. Standard errors from repeat experiments are shown.

When the same mutated FEN1s detailed above were employed, K93A most closely resembled the behavior of the WT protein with SF_+1−1_ (Fig. 5*B*)_._ Both R100A-Ca^2+^ and K93A/R100A-Ca^2+^ also reduced the ECCD signal of SF_+1−1_, although not to the same extent (Fig. 5*B*). However, Y40A, L130P, and D181A did not significantly alter the signal with SF_+1−1_ at 326 nm ±Ca^2+^. With double flap substrates and identically positioned 2APs (DF_+1−1_), R100A-Ca^2+^ and Y40A-Ca^2+^ both reduced the ECCD signal but not to the same extent as the WT protein in Ca^2+^ buffer (Fig. 4*D*).

When the 5′-phosphate was removed from the SF substrate (HO-SF_+1−1_) ECCD signals were significantly altered. A smaller decrease in ECCD signal at 326 nm was observed in the presence of divalent ions and WT protein relative to the same sample in EDTA (Fig. 5*C*). Moreover, the maximum of the signal with hFEN1-Ca^2+^ was blue-shifted relative to free HO-SF_+1−1_. When mutated hFEN1s interacted with HO-SF_+1−1_, only K93A was able to mimic the small change of WT hFEN1-Ca^2+^ with other proteins producing negligible effects within error.

**FIGURE 6.**
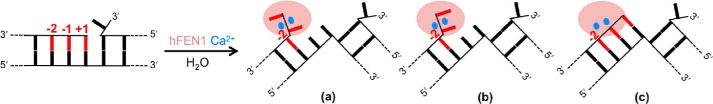
**Schematic model summarizing the responses of hFEN1-substrate complexes to addition of divalent metal ions based on ECCD results.** *Part (a)*, in the presence of divalent ions, unmodified substrates interacting with WT and K93A hFEN1s adopt an orientation of the −1 and −2 nt that is unstacked consistent with unpaired DNA. Also, stacking between the −1 and +1 nt is substantially reduced, suggesting control of their relative positions after unpairing. This observed conformational ordering of nucleobases is presumed to effect optimal contact between the scissile bond and active site metal ions and catalytic residues. *Part (b)*, a divalent metal ion-induced substrate state where there is a gross change in the orientation of the −1 and −2 nt suggestive of local DNA unpairing is adopted by R100A, D181A, and Y40A with unmodified substrates and by all proteins (except L130P) with substrates lacking a 5′-phosphate. In these cases, however, there is evidence that stacking reminiscent of ssDNA remains between the −1 and +1 nt, suggesting an unpaired DNA state that is not optimally positioned for reaction. *Part (c)*, the L130P mutation, modifications of the substrate that prevent accommodation of the 5′-flap under the helical cap (*i.e.* streptavidin conjugation to terminus of 5′-flap), or a mismatch at the +1 position prevent a DNA conformational change on addition of divalent ions. In these cases, the substrate is assumed to remain base-paired.

Combined results imply that changes in the relative orientation of the +1 and −1 nt occur consistent with reduced stacking of these nucleobases once unpaired and extrahelical. These changes evidently require the presence of the +1 5′-phosphate, Tyr^40^, Arg^100^, and Asp^181^ (Fig. 6*A*). We presume this reflects a conformation of the unpaired substrate that allows optimal orientation of the scissile phosphate relative to active site metal ions, basic residues, and attacking hydroxide. However, changes involving the −1 and −2 nt do not require these substrate and protein features, suggesting that in addition to requirements to effect unpairing of the substrate, additional residues are important to optimally position the unpaired DNA for reaction. (Fig. 6*B*). In contrast, perturbation of the secondary structure of the helical cap (L130P), prevention of substrate threading with a 5′-streptavidin block, or the inclusion of a +1 mismatch abolishes the ability of the protein-substrate complex to undergo the usual local DNA conformational changes when divalent metal ions are added (Fig. 6*C*).

## Discussion

Selection of both the correct DNA substrate and the correct phosphate diester bond for hydrolysis are key to hFEN1 biological function during replication and repair. Incorrect hydrolysis by hFEN1 would endanger genome integrity and necessitate the action of DNA repair mechanisms. The data presented here begin to reveal the details, interrelationships, and complexity of this process. The DNA junction itself is first recognized by its ability to bend 100°. This bent substrate conformation allows recognition of a single-nt 3′-flap and places the 5′-end of the reacting duplex close to the hFEN1 active site. However, the FRET results presented here demonstrate that junction bending does not require the 5′-portion of substrates to be accommodated by the protein either by threading 5′-flaps under the helical cap or by transfer to the active site metal ions (Fig. 2). Substrates that cannot transfer to the active site because metal ions are not present are still bent when bound to hFEN1 protein. Similarly, substrates that lack a 5′-flap or where the 5′-flap is prevented from threading underneath the helical cap are also bent, albeit with modestly reduced stability in the case of the streptavidin-blocked substrate. Thus, although global DNA bending must precede the local DNA conformational change necessary for reaction, it is not required to occur concomitantly with this process.

The key process in enforcing hFEN1 reaction site specificity is the transfer of the scissile phosphate diester located one nt into the reacting duplex onto active site metal ions. ECCD of (2AP)_2_ containing DNAs demonstrates that FEN1 substrates do not require a 5′-flap to enable this change (Fig. 3), underscoring the fact that exonucleolytic and endonucleolytic reactions of FEN1 substrates proceed by a common mechanism. However, DNAs with a mismatch at the end of the reacting duplex of the substrate are deficient in local DNA conformational changes (Figs. 4*B* and 6*C*). Similarly, the status of the 5′-termini of 5′-flaps is a determinant of the ability to bring about local DNA conformational change. Notably, the DNA substrate cannot position for reaction when the protein cannot properly accommodate 5′-flaps, as demonstrated by 5′-streptavidin blocking (Figs. 4*A* and 6*C*). Thus, when 5′-flaps with bound protein (*e.g.* RPA) or lacking free 5′-termini (continuous DNA of template strand) are encountered, reaction is prevented because the scissile phosphodiester bond cannot access the active site.

Alongside a requirement for threading of 5′-flaps demonstrated here, earlier work examining changes in orientation of the −1 and −2 nt in a (2AP)_2_ DF substrate concluded that individual conserved residues of the hFEN1 protein played little part in this DNA conformational change. However, the presence of active site divalent metal ions and the intact structure of the helical cap were essential for this reorientation (12, 17). Here, we show that this is also the case with exonucleolytic substrates lacking a 5′-flap and that a 5′-phosphate is not required for this −1 and −2 substrate distortion in these SF substrates (Fig. 3). The orientation of the +1 and −1 nt is also dependent on an intact helical cap and the presence of active site divalent metal ions (Figs. 4, *C* and *D*, and 5). However, the local conformational changes that occur with +1 and −1 nt are markedly altered by changes in both the substrate and protein.

Despite clear evidence of hFEN1-Ca^2+^ reorientation of the −1 and −2 nt when SF substrates lack a 5′-phosphate (Fig. 3*C*), only a small change is observed in the +1 and −1 ECCD signal (Fig. 5*C*). Assuming that the position adopted by the substrate in the presence of hFEN1-Ca^2+^ reflects the catalytically viable conformation, the 5′-phosphate monoester of SF substrates must form a key interaction required to assemble this state. Contacts to the 5′-phosphate monoester are also implied from the FRET studies (Fig. 2*C*), and although the substrate could still adopt the bent state, removal of the 5′-phosphate monoester of the SF substrate (HO-SF) increased the magnitude of *K*_bend_ substantially in Ca^2+^ buffer. With DF substrates, interactions with the equivalent 5′-phosphate diester (+1 position, *i.e.* the next phosphate 5′ in the chain to the scissile phosphate) presumably also play a key role in productive substrate positioning. This would explain earlier work demonstrating that neutralization of the charge of this +1 5′-phosphate by conversion to methyl phosphonate is detrimental to reaction (21). Thus, both ECCD and FRET behaviors reported here are consistent with earlier work in suggesting key interactions involving the substrate 5′-phosphate monoester/diester when DNA is positioned to react within the active site.

The mutation of conserved residues did not produce any substantive variation in the value of *K*_bend_ in the presence of Ca^2+^ (Fig. 2*C*). However, several of these residues were implicated in active site substrate positioning by studies of the +1 and −1 ECCD signal (Figs. 5*B* and 6). When the hFEN1 protein was altered to Y40A, there was no change in +1 and −1 ECCD signal in the presence of divalent metal ions compared with their absence with SF substrate and a substantially reduced effect with DF substrate compared with that seen with WT protein (Fig. 4*D*). Because Tyr^40^ forms stacking interactions with either the +1 or −1 nucleobases in substrate and product structures, respectively, these interactions are likely in the catalytically competent state. Previous fluorescence studies have revealed evidence for unusually fast quenching of substrate 2APs at both the +1 or −1 positions when bound to hFEN1-Ca^2+^, consistent with an interaction with Tyr^40^ (17). This was interpreted as an equilibrium between paired and unpaired forms of the substrate with Tyr^40^ interacting with the 2AP at +1 in paired and −1 in unpaired conformations. The data presented here support the idea that the Tyr^40^ residue plays an important role in optimal substrate positioning, and its mutation to alanine was found to reduce the rate of cleavage of DF substrate by a factor of 100.

There was also no change in +1 and −1 ECCD signal with D181A-Ca^2+^, and the lack of reorientation of the nucleobases in this instance may be related to metal ion positioning in the mutated protein (because Asp^181^ is directly coordinated to one of the active site M^2+^ ions). In addition, Arg^100^ appears to play a role in reorientation of the +1 and −1 nt because with this mutant the ECCD signal was reduced in the presence of Ca^2+^, but to a lesser extent than with WT hFEN1. Because the Arg^100^ residue contacts the cleaved phosphate monoester in product structures, it may well position the scissile phosphate diester in active site positioned substrate complexes. In contrast, Lys^93^ does not play a role in substrate positioning, and the impact of its mutation to alanine seems to be entirely related to catalysis (23).

Overall, these studies unravel the interrelationships between events in the hFEN1 catalytic cycle. Global DNA bending involving interactions with the duplex regions of substrates is essential to position the reacting duplex close to the active site. This facilitates accommodation of the 5′-flap (when present) and the local DNA conformational change required for reaction, but neither of these events is a prerequisite for the initial DNA interaction, suggesting that they occur after binding the substrate duplex regions. Once substrate is bound in a bent conformation, 5′-flaps, if present, are threaded underneath the cap. Threading is a prerequisite for transfer of the scissile phosphodiester to the active site in double flap substrates. Finally, the substrate adopts a single-stranded catalytically competent conformation traveling through the helical gateway (base of α4 and α2) contacting active site metal ions. ECCD results with −1 and −2 (2AP)_2_ substrates show that metal ions are sufficient to draw the substrate toward the active site providing the cap can adopt a helical state and that 5′-flaps can be threaded (Fig. 6*B*). However, ECCD data with +1 and −1 (2AP)_2_ DNAs demonstrate that the precise positioning of substrate is dependent on interaction with Tyr^40^ and Arg^100^ residues of the helical gateway and requires the presence of active site Asp^181^ and contacts to +1 phosphate of substrate (Fig. 6*A*).

## Author Contributions

T. D. C. and L. D. F. designed the FRET experiments, which were performed by S. I. A. and I. A. B. J. A. G., L. D. F., S. I. A., and J. C. E. designed the ECCD experiments, which were performed by S. I. A., J. C. E., M. J. T., V. J. B. G., and S. J. S. The proteins were purified by M. J. T., L. D. F., and J. C. E. All authors analyzed the data and contributed to preparation of the manuscript.

## Footnotes

4 The abbreviations used are:

FEN1: flap endonuclease-1
nt: nucleotide
2AP: 2-aminopurine
DF: double flap
SF: single (3′) flap
NL: nonlabeled
DOL: donor-only labeled
DAL: doubly labeled
ECCD: exciton coupled CD
h: human
TAMRA: tetramethylrhodamine
MMDF: mismatch containing (+1) double flap
SADF: 5′-streptavidin double flap.
